# Leptospirosis Causing Severe Acute Kidney Injury, Hyperbilirubinemia, and Thrombocytopenia: A Case Report

**DOI:** 10.7759/cureus.74854

**Published:** 2024-11-30

**Authors:** Jacob McElroy, Berk Celik, Yagnapriya Ammakola, Tannoz Norouzi, Tawhida Khatoon

**Affiliations:** 1 Internal Medicine, Lake Erie College of Osteopathic Medicine, Bradenton, USA; 2 Internal Medicine, Corewell Health, Royal Oak, USA; 3 Medicine, Oakland University William Beaumont School of Medicine, Rochester, USA

**Keywords:** : acute kidney injury, complications of leptospirosis, direct hyperbilirubinemia, severe leptospirosis, severe thrombocytopenia, thrombocytopenia, weils' disease

## Abstract

Leptospirosis, an infection caused by the spirochete Leptospira and commonly attributed to the underdeveloped world, is frequently under-diagnosed in the United States. This report discusses the case of a 79-year-old male with no significant medical history who presented to the ED with recurrent falls. Initial laboratory results demonstrated severe acute kidney injury, hyperbilirubinemia, and thrombocytopenia. Leptospirosis can mimic a wide variety of medical conditions, ranging from acalculous cholecystitis to alcoholic cirrhosis. Diagnosing leptospirosis requires an in-depth and detailed patient history. This case emphasizes the need for heightened awareness among healthcare providers to consider leptospirosis in their differential diagnoses.

## Introduction

Leptospirosis, caused by the spirochete bacterium Leptospira, is considered to be the most common zoonotic infection in the world. Infections are transmitted through direct exposure to urine from infected animals or by inoculation of contaminated water or soil [1]. Leptospirosis is found globally, primarily in tropical or subtropical regions with humid environments, and is associated with travelers returning from these regions [1]. Worldwide annual estimates include 1.03 million cases and 58,900 deaths [1]. Infected animals can carry the disease without showing signs or symptoms. Commonly infected animals range from farm animals like cattle and pigs to wild animals such as rats, raccoons, and domesticated pets like dogs [2]. In the United States, 100 to 200 cases are reported annually; however, it is thought that leptospirosis is under-diagnosed, given its susceptibility to a broad range of antibiotics. Leptospirosis can present in two distinct clinical syndromes: icteric or anicteric. The icteric phase, known as Weil’s disease, can present with signs of fever, renal failure, hemorrhage, and respiratory distress [2]. The most common clinical manifestation in humans is a flu-like illness with mild hepatic and renal impairment [3]. However, the icteric phase can present with hepatorenal failure, encephalopathy, and hemorrhage [4]. Here, we present a case of leptospirosis that presented with encephalopathy, severe thrombocytopenia, jaundice, and acute kidney injury.

## Case presentation

A 79-year-old male with no significant past medical history presented after experiencing multiple falls. Most recently, he was found on the floor by a neighbor and was estimated to have been there for nine hours. Physically, the patient was diffusely jaundiced, icteric, confused, and demonstrated poor hygiene. An abdominal ultrasound showed mildly increased echogenicity in the liver and a thickened gallbladder with a moderate amount of sludge, concerning for acalculous cholecystitis. Further evaluation with a hepatobiliary iminodiacetic acid (HIDA) scan showed no visualization of the gallbladder. Subsequent magnetic resonance cholangiopancreatography (MRCP) findings were consistent with acute cholecystitis and revealed no evidence of cirrhosis. Pertinent laboratory findings on admission included: blood urea nitrogen (BUN) 120 mg/dL, creatinine (Cr) 2.20 mg/dL, alanine aminotransferase (ALT) 68 IU/L, aspartate aminotransferase (AST) 103 IU/L, albumin 3.1 g/dL, total bilirubin 5.3 mg/dL, WBC 2.7 x10^3^/µL, red blood cells (RBC) 4.05 x 10^6^/µL, hemoglobin (Hgb) 12.5 g/dL, platelets (PLT) 31 x10^3^/µL, and creatine kinase (CK) 1,168 IU/L. An infectious panel for Epstein-Barr virus (EBV), cytomegalovirus (CMV), HIV, and hepatitis were all negative. Given that he lived alone, potential alcohol abuse was investigated but was ruled out by non-cirrhotic liver findings on MRCP and a negative phosphatidylethanol (PEth). The patient was started on a continuous lactated Ringer's (LR) solution at 125 ml/hr, IV Zosyn, lactulose 20 g q6 hrs, rifaximin 550 mg bid, IV albumin infusion 25 g tid, and IV Protonix 40 mg bid. Thiamine, folate, and vitamin B12 were supplemented as the patient was found to be severely deficient.

During his admission, the patient developed bilateral deep venous thrombosis (DVT) and a ventilation-perfusion (V/Q) scan demonstrated perfusion irregularities concerning for a pulmonary embolism. Interventional radiology (IR) placed an inferior vena cava (IVC) filter as heparin was contraindicated given the patient's severe thrombocytopenia. Over the course of the next three days, BUN increased to 156 mg/dL, Cr to 5.91 mg/dL, total bilirubin to 23.6 mg/dL, direct bilirubin to over 15 mg/dL, and platelets dropped to 19 x10^3^/µL. Hematology decided to start intravenous immunoglobulin (IVIG) therapy, and the patient was given two doses during his admission. Hepatology and general surgery recommended that a percutaneous cholecystostomy (PCT) drain be placed, but IR believed that imaging was not consistent with acalculous cholecystitis and that PCT placement was not necessary.

Upon further questioning about the patient’s living circumstances, his daughter revealed that he lived in an unhygienic environment with three dogs and two chickens. The patient had remained on IV Zosyn throughout his hospital stay, and the internal medicine team decided to work up possible infectious, zoonotic etiologies. On day five of the patient’s admission, labs suddenly began to improve and leptospirosis IgM antibodies were ordered. Antibody titers came back positive, and labs on discharge revealed a BUN of 26 mg/dL, Cr of 1.29 mg/dL, AST of 51 IU/L, ALT of 45 IU/L, total bilirubin of 8.2 mg/dL, direct bilirubin of 6.1 mg/dL, and platelets of 239 x10^3^/µL. Laboratory findings during his admission are summarized in Table 1 below. Infectious disease determined that the patient had received adequate antibiotic coverage and that he was cleared for discharge. The patient’s children were informed that his dogs would need to be taken to the veterinarian for treatment, and they began the process of arranging more sanitary, supervised living accommodations.

**Table 1:** Laboratory progression during admission. Hgb: Hemoglobin; BUN: Blood Urea Nitrogen; ALT: Alanine Aminotransferase; AST: Aspartate Aminotransferase.

	Reference Range	Admission	Day 3	Discharge
RBC	4.35-5.65 (x 10^6^/µL)	4.05	3.51	3.72
WBC	4.5-11.0 (x10^3^/µL)	2.7	9.9	11.4
Hgb	13.5-17.5 (g/dL)	12.80	10.9	11.5
Platelets	150-450 (x10^3^/µL)	31	19	239
BUN	6-24 (mg/dL)	120	156	26
Creatinine	0.7-1.3 (mg/dL)	2.20	5.91	1.29
ALT	4-36 (IU/L)	68	55	45
AST	8-33 (IU/L)	103	81	51
Albumin	3.4-5.4 (g/dL)	3.10	1.87	3.10
Creatinine Kinase	55-170 (U/L)	1,168	571	50
Bilirubin, Total	0.1-1.2 (mg/dL)	5.3	23.6	8.2
Bilirubin, Direct	0.0-0.3 (mg/dL)	-	> 15	6.1

## Discussion

Leptospirosis is predominantly seen in tropical and subtropical regions. It is a zoonotic disease, spreading through exposure to animals like rodents and dogs. In this patient, contaminated urine from his dogs was the source of Leptospira. Leptospirosis can mimic many other diseases, highlighting the importance of taking a thorough occupational and sanitary history in patients [5]. According to the CDC, only 100-150 cases are reported annually in the United States [6]. Anicteric leptospirosis accounts for ninety percent of cases and presents as a non-specific flu-like illness that is self-limited [2]. Weil's syndrome, a severe icteric form of leptospirosis, accounts for 5-10% of cases and can present with symptoms of fever, rigors, myalgias, and headache in most patients [7]. Persistent or worsening infection can lead to the immunological phase, resulting in significant organ damage [1]. Subsequently, the patient may develop renal failure, jaundice, cardiac dysrhythmias, aseptic meningitis, conjunctival injection (with or without hemorrhage), ocular pain, myalgia, lymphadenopathy, and hepatosplenomegaly. Muscle tenderness, rigidity, splenomegaly, pharyngitis, abnormal respiratory auscultation, skin rash, or hepatomegaly occur in 7% to 40% of cases [8]. Other serious complications include myocarditis, uveitis, acute respiratory distress syndrome, rhabdomyolysis, and pulmonary hemorrhage, which can be fatal.

Complications are often due to vasculitis and endothelial cell injury, with a cytokine storm involving IL-6, TNF alpha, and IL-10 contributing to multi-organ failure [9]. Laboratory findings in severe leptospirosis can include hyperbilirubinemia, anemia, thrombocytopenia, elevated creatinine, and hyponatremia [10]. In some cases, bilirubin levels can reach up to 30, due to hepatocellular damage and leakage of bilirubin from bile ducts [11]. The diagnosis of leptospirosis is usually made by serological tests after other etiologies have been ruled out. In our case, IgM antibodies against Leptospira were ordered and used to make a diagnosis for our patient. Leptospira is sensitive to a broad range of antibiotics, including doxycycline, penicillin, third-generation cephalosporins, and others [11]. We were covering our patient with piperacillin and tazobactam for suspected acalculous cholecystitis. This was the only intervention that produced clinical improvement, which made us suspect an infectious etiology. Although not the most commonly used treatment, leptospirosis in our patient was susceptible to treatment with piperacillin and tazobactam (Zosyn). Populations at higher risk of leptospirosis exposure include economically disadvantaged communities, individuals living in poor sanitary conditions, those in contact with infected rodents and dogs, and people exposed to contaminated freshwater, such as triathletes [12]. Dogs serve as a reservoir for Leptospira, and exposure rates are increasing in the United States due to canine contact and the reluctance of dog owners to vaccinate against leptospirosis [13]. Treating dogs with a quadrivalent vaccine against leptospirosis has been shown to reduce incidence in highly endemic areas, suggesting the need for vaccination of domesticated canines [13]. Our patient was instructed to take his dogs to the veterinarian for vaccination and prevention of repeat exposure.

## Conclusions

Leptospirosis is a diagnosis that requires an accurate patient history and high clinical suspicion after ruling out other differential diagnoses. Upon arrival, our patient was visibly unhygienic, prompting investigative questioning that helped us obtain clues leading to a diagnosis. Although easily treatable, misdiagnosing patients with leptospirosis can lead to serious complications and adverse outcomes. With the prevalence of leptospirosis increasing across the country, we hope this case report emphasizes the importance of thinking outside the box and considering leptospirosis in the setting of acute kidney injury, thrombocytopenia, and hyperbilirubinemia.
